# The importance of work conditions and health for voluntary job mobility: a two-year follow-up

**DOI:** 10.1186/1471-2458-12-682

**Published:** 2012-08-21

**Authors:** Cathrine Reineholm, Maria Gustavsson, Mats Liljegren, Kerstin Ekberg

**Affiliations:** 1 HELIX VINN Excellence Centre, Linköping University, 581 83, Linköping, Sweden; 2 National Centre for Work and Rehabilitation, Department of Medical and Health Sciences, Linköping University, 581 83, Linköping, Sweden; 3 Department of Behavioural Sciences and Learning, Linköping University, 581 83, Linköping, Sweden; 4 Department of Law, Psychology and Social Work, Örebro University, 701 82, Örebro, Sweden

**Keywords:** Work conditions, Health, Burnout, Voluntary job mobility, Two-year follow-up

## Abstract

**Background:**

Changing jobs is part of modern working life. Within occupational health, job mobility has mainly been studied in terms of employees’ intentions to leave their jobs. In contrast to actual turnover, turnover intentions are not definite and only reflect the probability that an individual will change job. The aim of this study was to determine what work conditions predict voluntary job mobility and to examine if good health or burnout predicts voluntary job mobility.

**Methods:**

The study was based on questionnaire data from 792 civil servants. The data were analysed using logistic regressions.

**Results:**

Low variety and high autonomy were associated with increased voluntary job mobility. However, the associations between health and voluntary job mobility did not reach significance. Possible explanations for the null results may be that the population was homogeneous, and that the instruments for measuring global health are too coarse for a healthy, working population.

**Conclusions:**

Voluntary job mobility may be predicted by high autonomy and low variety. The former may reflect that individuals with high autonomy have stronger career development motives; the latter may reflect the fact that low variety leads to job dissatisfaction. In contrast to our results on job content, global health measurements are not strong predictors of voluntary job mobility. This may be because good health affects job mobility through several offsetting channels, involving the resources and ability to seek a new job. Future work should use more detailed measurements of health or examine other work settings so that we may learn more about which of the offsetting effects of health dominate in different contexts.

## Background

Since the 1990s, working life has undergone several changes. As a result of globalization, new technology, and a gradual shift from production to service jobs, new work tasks and new types of jobs have evolved [1]. Today, changing jobs is part of modern working life. In the literature, a number of concepts have been used for defining this, such as turnover, job change, job separation and job mobility. In this study, the focus is on employees changing jobs voluntarily, defined as voluntary job mobility.

Bad work conditions increase peoples’ willingness to change jobs, but it seems that the decision to actually change jobs is more complex and depends on several factors. Poor health may lead to downward mobility or redundancy, but it is also suggested that poor health increases the risk of being “locked in”, i.e. non-mobility. Changing jobs seems to lead to increased job satisfaction and increased health, but good health may also be a condition for having the ability or strength to actually change jobs. Given this background, the objective of the present study is twofold: 1) to determine what work conditions predict voluntary job mobility and 2) to examine if good health or burnout predicts voluntary job mobility.

### Voluntary job mobility and work conditions

Within occupational health, voluntary job mobility has mainly been studied in terms of the intention of employees’ to leave their jobs. In contrast to actual job mobility, turnover intentions are not definite. Intention to leave is an attitude that reflects the individual’s propensity to change jobs [2,3]. High turnover intentions are related to negative factors at work, such as high work load [4], job dissatisfaction [5], and limited opportunities for advancement [6]: i.e., bad work conditions seem to affect peoples’ willingness to change jobs. For example, Brannon, Barry, Kemper, Schreiner, and Vasey [7] found that low skill variety was related to an increased intention to leave the job. Other studies have found associations between high turnover intentions and low autonomy, low feedback [8,9], and a low level of social support [10]. Although turnover intentions are often considered to be a strong predictor of future job mobility [3,11,12], a direct association between turnover intentions and job mobility is only weakly supported, possibly because of the cross-sectional design of most studies.

Changing jobs increases job satisfaction [13] and health [14], but few studies have investigated causes of actual job mobility. In a study by Jaros [12], job satisfaction and organizational commitment were found to be negatively related to voluntary job mobility. Among nurses leaving their jobs, Skytt, Ljunggren, and Carlsson [15] found that lack of social support from supervisors and heads of department was a common reason for leaving, and changing jobs increased job satisfaction. The traditional view is that people change jobs due to dissatisfaction [16], and Castle and Engberg [17] claim that the decision to quit a job consists of cognitive stages where job dissatisfaction is the initial state. In addition to job dissatisfaction, Mobley, Griffeth, Hand, and Megliano [18] suggest that job mobility may also be due to the opportunity to change to a more attractive job. According to Mitchell, Holtom, Lee, Sablynski and Erez [16], the combination of job attitudes and job alternatives predicts intention to leave, and if an alternative job is better than their current one, people will change. Thus, according to present knowledge, changing jobs may increase job satisfaction, but knowledge is essentially lacking concerning what work conditions predict voluntary job mobility in a longitudinal perspective.

Although job mobility is often seen as an opportunity to find a better job [13], employees do not always choose to quit even if they are dissatisfied with their present job and have the opportunity to change. This means that additional factors may affect job mobility and the relationship between turnover intentions and job mobility may vary [2]. Thus, job dissatisfaction does not necessarily lead to actual job mobility [19]. Individual characteristics such as age, education level and having a family are well-known indicators associated with job mobility [20-22]. Having a family is also negatively related to job seeking behaviour, as found by van Hooft, Born, Taris, van der Flier and Blonk [23]. These factors should therefore be taken into consideration when studying voluntary job mobility. Naturally, opportunities for changing jobs are dependent on additional factors such as recession, high unemployment rates [24], and geographic factors [25].

### Voluntary job mobility and health

Voluntary job mobility seems to improve job satisfaction [13,15] and reduce physical and mental strain [26,27], possibly because this type of mobility may involve career development or positive choices. Poor health, on the other hand, may increase the risk of downward mobility to lower qualified jobs and unhealthy employees are also more likely to be redundant than healthy employees [28-30]. At the structural level, low mobility in the labour market may be a possible explanation for ill health and long-term sick leave [31], due to a mismatch between job demands and individual capacity. However, a low degree of job mobility is not necessarily predictive of job satisfaction or a healthy organizational environment, as pointed out by Strolin-Goltzman [32].

Compared with other countries, Sweden has low job mobility and a possible explanation may be found in the Swedish labour market regulations [31]. The Employment Protection Law [33] protects employees with long employment tenure from being laid off. According to von Otter [34], the law may also lock people into permanent but not preferred jobs because it makes employees hesitate to change jobs due to the risk of being first in line to be redundant at the new workplace due to short work tenure. Low job mobility may also increase the risk of becoming embedded or in a locked-in position, which increases the risk of ill health [35,36].

## Methods

### Materials

A questionnaire was sent by post to all employees (*N* = 1010), including those on sick leave and on leave of absence, at three different regional organizations of the Swedish National Labour Market Administration (AMV). Of the 1010 employees, 602 (60%) were women and 408 (40%) were men. The average age was 48.7 years (SD 9.28 years), ranging from 25 to 65 years, and most of the respondents were working as employment officers in different local employment agencies. A total of 792 employees (78%) responded to the questionnaire. Forty-five percent of the respondents had a university degree and 43% had graduated from upper secondary school. Most (80%) were married or living with a partner and 46% had children living at home.

Information regarding job mobility for the two years after the baseline questionnaire was provided by the organization where the respondents were employed. Respondents who had retired between baseline and the follow-up (*n* = 15) were excluded from the analysis.

### Ethics

Ethical principles for social science have been observed, in that the purpose of the research was explained, informed consent was received, confidentiality was maintained, no individual response could be recognized etc. The questionnaires were sent by post to each individual; they were returned by the respondent in an enclosed response envelope and were only read by the researchers.

The study was approved by the Ethics Committee at Linkoping University.

### Measurements

#### Individual characteristics

Sex, age, education level, civil status, and having children living at home were used as demographic variables.

#### Work conditions

Work conditions were measured by the Job Characteristic Inventory (JCI) [37]. The JCI was developed to measure how job characteristics relate to productivity and job satisfaction in different organizations. Variety, autonomy, task identity, and feedback are suggested as core dimensions because employees will be able to obtain satisfaction and perform well if they experience variation in work tasks, can plan and decide how work should be carried out, can identify the results of their efforts, and get feedback on how they are performing. One example item for measuring variety is: “How much variety is there in your job?” The response options on a 5-point Likert scale range from *very little* (1) to *very much* (5). Cronbach’s alpha was 0.84 for the variety scale, 0.82 for the feedback scale, 0.79 for the task identity scale, and 0.69 for the autonomy scale.

#### Health

Measurements of health were chosen to capture a range of good health to bad health. The SF-36 [38] is a generic instrument designed to be applicable to a wide range of physical and mental health conditions. Vitality, as a component of good health, was measured by the vitality scale from the SF-36. The vitality scale captures health states ranging from feeling tired and worn out to feeling full of pep and energy. One example item for measuring vitality is: “How much of the time during the four past weeks did you have a lot of energy?” The response options on an 8-point Likert scale range from *all of the time* (1) to *none of the time* (8). Cronbach’s alpha was 0.85 for the vitality scale.

Overall health was measured by the Visual Analog Scale from the EuroQol instrument (EQ-VAS) [39]. The purpose of the self-rating scale is to capture overall health; the respondents rate their current physical and mental health state ranging from *the worst state you can imagine* (0) to *the best state you can imagine* (100).

#### Burnout

The Copenhagen Burnout Inventory (CBI) was developed to measure burnout, anxiety, and fatigue [40]. Only the generic part of the CBI, personal burnout, was used in the present study as an indicator of general burnout. One example item for measuring burnout is: “How often do you feel worn out?” and the response options on a 5-point Likert scale range from *always* (1) to *never/almost never* (5). The scale ranges from 0 to 100, where the first category, *always*, is scored 100 and the fifth category, *never/almost never*, is scored 0. Cronbach’s alpha was 0.90 for the personal burnout scale.

#### Voluntary job mobility

Voluntary job mobility was defined as voluntarily changing jobs, i.e. leaving the organization. Information about job mobility between baseline and two years later was provided by the organization where the respondents were employed. Voluntary job mobility was coded as non-mobile (still at original workplace or internal mobility) or mobile (changing organization/employer).

### Statistical analysis

Mobility and non-mobility were examined with cross-tabulation and the chi-squared test. The distribution of the means and standard deviations for work conditions and self-rated health and burnout in relation to non-mobility and mobility were calculated using the *t*-test. To investigate how demographic variables, work conditions and health predict voluntary job mobility, logistic regressions were performed. The results are presented as odds ratios (OR) with 95% confidence intervals (CI). SPSS version 17.0 was used for the statistical analyses.

## Results

### Non-response

The response rate was 78%. Non-responders and dropouts were analysed with the available data (sex and age). The response rate did not differ significantly between the sexes. The responders were older than the non-responders (*p* < .01).

### Individual characteristics

Voluntary job mobility between baseline and the two-year follow-up distributed for sex, age, education, civil status, and having children living at home are presented in Table 1. During the study period, 872 subjects (88%) remained at the same workplace (i.e. they were non-mobile) and 122 (12%) subjects changed organization (i.e. they were mobile). Younger respondents were more mobile than older respondents (*p* < .01). There were no significant differences regarding sex, education level, civil status, and having children living at home.

**Table 1:** **Descriptive statistics for voluntary job mobility between baseline and the two-year follow-up distributed among sex, age, education level, civil status, and having children living at home (*N*** **= 1010)**

		**Non-mobile,*n*(%)**	**Mobile,*n*(%)**	***p***
Sex	Women	521 (60)	72 (59)	.87
	Men	351 (40)	50 (41)	
Age	Under 35years	76 (9)	21 (17)	<.001
	35–44years	194 (22)	39 (32)	
	45–54years	308 (35)	34 (28)	
	55years and older	294 (34)	28 (23)	
Education	9-years compulsory school	64 (8)	10 (11)	.07
	2years upper secondary school	174 (22)	17 (18)	
	3/4years upper secondary school	168 (22)	17 (18)	
	University	358 (46)	45 (47)	
	Other	15 (2)	6 (6)	
Civil status	Single	115 (17)	18 (21)	.53
	Cohabitee/married	538 (80)	69 (78)	
	Other	20 (3)	1 (1)	
Children	No	353 (53)	50 (57)	.49
	Yes	314 (47)	38 (43)	

Descriptive statistics (means and standard deviations) for work conditions and self-rated health and burnout among the non-mobile and mobile employees are presented in Table 2. Individuals who left the organization rated their autonomy in the job they left as higher than non-mobile individuals did (*p* < .05). There were no differences between the groups regarding the following work conditions: variety, feedback, and task identity. Self-rated health and burnout did not differ between the mobile and the non-mobile groups.

**Table 2:** **Descriptive statistics (means and standard deviations) and results of*t*-test for work conditions and health distributed among non-mobile and mobile**

	**Non-mobile (*n*=872)**	**Mobile (*n=*122)**	***p***
*Work conditions*			
Variety	3.35 (0.67)	3.26 (0.72)	.24
Autonomy	3.69 (0.57)	3.83 (0.59)	.03
Feedback	2.78 (0.73)	2.84 (0.80)	.54
Task identity	3.70 (0.75)	3.84 (0.67)	.09
*Health*			
Vitality	59.2 (23.1)	60.4 (21.9)	.66
VAS	73.3 (18.6)	74.3 (18.6)	.64
Burnout	44.1 (19.2)	44.8 (18.5)	.71

### Work conditions, health, burnout, and voluntary job mobility

Logistic regression analyses were used to study associations between work conditions, health, burnout, and voluntary job mobility. Individual characteristics (sex, age, education level, civil status, having children living at home) were controlled for (Table 3). Crude ORs were calculated for all variables to determine the association with voluntary job mobility. High autonomy (OR 1.55, CI 1.04–2.33) was associated with voluntary job mobility.

**Table 3:** **Associations between work conditions, health, burnout at baseline, and voluntary job mobility at follow-up (OR,*p*-value and 95% CI), controlled for sex, age, education, civil status and having children living at home**

	**Crude**^**a**^			**Model 1**^**b**^			**Model 2**^**c**^		
	**OR**	***p***	**95% CI**	**OR**	***p***	**95% CI**	**OR**	***p***	**95% CI**
*Work conditions*	*n*=748			*n=*727			*n*=724		
Variety	0.82	.24	0.59–1.14	0.62	.02	0.42–0.91	0.63	.02	0.42–0.94
Autonomy	1.55	.03	1.04–2.33	1.71	.05	1.00–2.89	1.79	.04	1.03–3.09
Feedback	1.10	.53	0.81–1.50	1.07	.69	0.76–1.51	1.22	.29	0.85–1.75
Task identity	1.32	.09	0.96–1.82	1.21	.36	0.83–1.82	1.21	.38	0.79–1.84
*Health and burnout*	*n*=731								
Vitality	1.00	.66	0.99–1.01				1.01	.46	0.99–1.03
VAS	1.00	.64	0.99–1.02				1.01	.42	0.99–1.03
Burnout	1.00	.71	0.99–1.01				1.02	.07	1.00–1.04

^a^Crude OR.

^b^Model 1: work condition variables, adjusted for each other.

^c^Model 2: Model 1 adjusted for health and burnout variables.

In Model 1, work conditions were adjusted for each other to determine the association with voluntary job mobility. Low variety (OR 0.62, CI 0.42–0.91) and high autonomy (OR 1.71, CI 1.00–2.89) were associated with voluntary job mobility.

In Model 2, the associations between work conditions and voluntary job mobility were adjusted for health variables. Low variety and high autonomy remained associated with voluntary job mobility. The associations between health and voluntary job mobility were not significant, but burnout was close to significance (OR 1.02, CI 1.00–1.04).

## Discussion

The purpose of this study was to examine what work conditions predict voluntary job mobility, and whether good health or burnout predicts voluntary job mobility. The respondents in this study had a high degree of job mobility. After two years, 12% had left their organization compared with the average workplace mobility in Sweden of 8% during the same period [22]. The study population was not only well-educated; in all likelihood they also had a good knowledge of the labour market, since they worked as employment officers. High employability and good opportunities for changing jobs may therefore be a possible explanation for the high degree of job mobility in the study population.

Younger individuals were more mobile. It can be assumed that trying different types of jobs or occupations while building up a career is more common among younger individuals. As individual characteristics are important for being able to change jobs [21], sex, age, education level, civil status, and having children living at home were controlled for in the analysis.

### Work conditions and voluntary job mobility

The results showed that work conditions were related to voluntary job mobility. Low variety (i.e. a low degree of variety in work tasks or procedures) was associated with high voluntary job mobility. According to the activation theory, stimulation by variety and complexity of tasks increases the activation level, which is suggested to improve motivation and job satisfaction [41]. Repetitive tasks may decrease motivation, job satisfaction, and performance [42,43]. Low variety has been associated with increased turnover intentions in several studies [7,8], and according to Castle and Engberg [17] job dissatisfaction is the first step towards the decision to change jobs. Thus, as high variety is related to job satisfaction [44], low variety may be assumed to predict voluntary job mobility due to job dissatisfaction.

Employees scoring high on autonomy, i.e. the extent to which employees have a major say in planning, performing, and controlling their work, had higher voluntary job mobility. In previous research, low autonomy was associated with high turnover intentions [8,9] and decreased job satisfaction [45]. It is reasonable to assume that respondents who scored high on perceived autonomy in this study changed jobs for other reasons than dissatisfaction with their current job, such as career development and advancement to higher skilled jobs. This confirms the statement by Mobley, Griffeth, Hand and Megliano [18], that in addition to job dissatisfaction, voluntary job mobility may be related to the decision to change to a more attractive job. According to the gravitational theory [27,46,47], people move to a job that matches their ability level, but some people may have higher goals and strive for advancement that matches their future goals and career plans. Changing jobs seems to be triggered by individual motives and people change jobs in the expectation that the new job will be an improvement on their current job, in terms of better work conditions, career development, etc. [27]. This may also gain support from the expectancy theory, which perceives individuals as rational beings who choose between action options in order to maximize outcomes and minimize costs [48], or to maximize pleasure or minimize pain [49]. The expectancy theory also proposes that individuals’ choices about a certain act depend on their beliefs in their own capabilities and the reward from it [50]. Thus, voluntary job mobility may be due to different reasons: job dissatisfaction but also career development and new challenges.

### Voluntary job mobility and health

Despite using instruments that were expected to capture the spectrum from good health to bad health, the associations between health and voluntary job mobility did not quite reach significance. One possible explanation for the null results is that the instruments for measuring global health are too coarse for a healthy, working population. Furthermore, they are also designed to capture symptoms. As the respondents in this study were all white-collar workers with no physically demanding work tasks, this may also have affected the null results.

Voluntary job mobility is most likely due to two forces for mobility: job dissatisfaction and career development. These forces may, in turn, define individuals who are able to act and mobilize themselves to a new job. According to a holistic approach to health, health is related to an individual’s ability to act, and an individual has full health if, in a given standard situation, he or she has the ability to fulfil vital goals [51]. Drawing on this health approach, work itself may be important for health, which, in turn, may be important for voluntary job mobility.

### Study limitations

A weakness of this study is the homogeneous population. The respondents were all well-educated white-collar workers and worked in the same organization with similar work tasks. This may have caused imprecise estimation of associations, compared with a more heterogeneous population.

The strength of this study is the two-year follow-up data and the high response rate.

## Conclusions

We find that voluntary job mobility is predicted by high autonomy and low variety. The former may reflect that individuals with high autonomy have stronger career development motives; the latter may reflect the fact that low variety leads to job dissatisfaction. In contrast to our results on job content, global health measurements are not strong predictors of voluntary job mobility. This may be because good health affects job mobility through several offsetting channels, involving the resources and ability to seek a new job. Future work should use more detailed measurements of health or examine other work settings so that we may learn more about which of the offsetting effects of health dominate in different contexts.

## Competing interests

The authors declare that they have no competing interests.

## Authors’ contributions

CR, MG, and KE designed the study. CR was responsible for writing the manuscript and performed all statistical analysis. ML collected the data. All authors have contributed to the analysis and interpretation of the findings, provided input on manuscript drafts, and approved the final manuscript.

## Pre-publication history

The pre-publication history for this paper can be accessed here:

http://www.biomedcentral.com/1471-2458/12/682/prepub
